# Surgical education through integration of robotic telesurgical technology and AI

**DOI:** 10.1002/ags3.12751

**Published:** 2023-10-31

**Authors:** Kenichi Hakamada

**Affiliations:** ^1^ Department of Gastroenterological Surgery Hirosaki University Graduate School of Medicine Hirosaki Japan

Robotic surgery technology is now fusing with ICT (information and communications technology) and AI (artificial intelligence) to bring about new changes in surgical education.

In robotic surgery, images of the surgical field are converted into digital signals by a 3D high‐definition camera and transmitted to the surgeon's viewer, and surgical operations are digitally converted into 3D coordinate axis information and acceleration information, which are transmitted to the robotic arm and accurately reproduced as forceps manipulations. Therefore, robotic surgery can be described as a digitization technology for surgery.

Robotic surgery enables surgery without motion restrictions under detailed images deep inside the body, and the number of surgeries, especially in the field of gastrointestinal surgery, is increasing due to expectations for precision. In this volume, McCarron et al. report that they started a robotic HPB program in 2008, performed 100% of Whipple procedures by 2020, and over 1600 complex hepatobiliary surgeries by robotic surgery to date. Unfortunately, at this time, there is still little evidence of a clinical long‐term outcome advantage of robotic surgery over laparoscopic surgery, but the digitization technology of surgery offers two major enhancements besides precision.

The first is telesurgery integrated with ICT. Robotic surgery began to be developed in the 1970s for use in outer space and on the battlefield. Later, as military technology was converted to civilian use, development progressed as surgical robots for general medical use. In 2001, the world's first cholecystectomy was performed by connecting New York and Strasbourg, France via a dedicated line, followed by 22 cases of abdominal surgery in Canada. All these early attempts were reportedly successful, but unacceptably long transmission delay times, high communication costs, inadequate communication security, and even the discontinuation of surgical robotics led to a setback in the development of telesurgery technology. However, recent advances in ICT, the development of high‐speed communication networks, and the development of new telesurgery‐compatible robots have led to a re‐evaluation of telerobotic surgery.

In Japan, following the legal establishment of telesurgery in 2019, the Japan Surgical Society has established a project team to conduct research on the social implementation of telesurgery. It was found that the transmission delay, which is the biggest barrier in implementing telesurgery, does not affect surgical operations as long as it is less than 100 ms.^1^ In 15 telesurgery experiments conducted using commercial lines by September 2023, the communication delay between two sites 100–700 km apart was 4–25 ms round‐trip, and the total delay time, including the video information processing time, was about 50 ms.^2^, ^3^, ^4^ In these remote surgical environments, we have performed several porcine gastrectomies, rectal resections, cholecystectomies, and nephrectomies using two surgical robots (Medicaroid's hinotori and Riverfield's Saroa) and confirmed that they can be performed safely. We have also confirmed that emergency repair of large vessel injuries is possible.^5^ Furthermore, cadaveric pyloric gastrectomy and total gastrectomy have been performed and confirmed to be as accurate as clinical operations.^6^ In addition, robotic companies have developed a dual cockpit for remote surgical guidance and an annotation device to remotely draw surgical operations on video, creating a new environment for surgical education using telesurgical technology.

A second extension of the digitization of surgery is its integration with AI. Although many surgeons are skeptical of surgical automation at this time, systems that use machine learning of data labeled by surgeons from excellent digitized surgical videos to segment surgical scenes and recognize structures such as gallbladders and blood vessels in surgical videos are already being used for surgical education on a commercial basis. In addition, there are high expectations for the use of the system as a surgical navigation system by accumulating and machine learning superior surgical operations. Digitalization of surgery is expected to contribute to the efficiency of the surgical education process.

On the other hand, surgical education centered on robotic surgery has its limitations. With the exception of a few robots, many robots still do not have tactile sensation. The human hand's sense of touch includes a variety of functions, including superficial (touch, pain, temperature) and deep (pressure, position, vibration, etc.) senses, as well as cortical senses (two‐point discrimination, three‐dimensional discrimination ability, etc.). Moreover, the number of sensors possessed by human hands far exceeds that of robots. It may be said that humans are still the better robots. However, the emergence of generative AI will accelerate the development of science and technology and may easily solve these problems. If this happens, the safety of robotic surgery will increase, and the effectiveness of surgical education using telesurgical technology will be further enhanced. Digitized surgery will significantly change the methods and quality of surgical education and enable surgical education beyond the limitations of space and distance.

## CONFLICT OF INTEREST STATEMENT

The author declares no conflicts of interest for this article.
